# The predictive role of social isolation in COVID-19 anxiety among older adults

**DOI:** 10.1186/s12877-025-06046-w

**Published:** 2025-05-26

**Authors:** Bahrami Mahdie, Marzieh Khatooni, Mojtaba Senmar, Hosseinali Movahedeh, Fateme Zarei, Seyedeh Ameneh Motalebi, Mohammad Amerzadeh

**Affiliations:** 1 Non-Communicable Diseases Research Center, Research Institute for Prevention of Non-Communicable Diseases, Qazvin University of Medical Science, Qazvin, Iran; 2 Department of Nursing, School of Nursing and Midwifery, Qazvin University of Medical Science, Qazvin, Iran

**Keywords:** Social isolation, Older adults, COVID-19 anxiety

## Abstract

**Background:**

COVID-19 is one of the most significant diseases that has spread in the present century. Older adults have been greatly affected by the negative consequences of the COVID-19 pandemic. Social isolation is one of the challenges of modern aging that leads to various detrimental conditions for health. Therefore, this study aimed to determine the predictive role of social isolation in COVID-19 anxiety among older adults.

**Methods:**

This cross-sectional study with a descriptive-analytical approach was conducted in 2023. A total of 300 older adults were included in the study through cluster sampling from three districts in Qazvin City, Iran based on inclusion criteria. Data collection involved demographic information questionnaires, the COVID-19 Anxiety Questionnaire, and the Social Isolation Questionnaire. Data analysis was performed using descriptive tests and multivariate regression analysis with the SPSS 23 software.

**Results:**

The mean and standard deviation of COVID-19 anxiety and social isolation among the participants were 10.99 and 28.10, respectively. The results of the multivariable regression model indicated that marital status (*P* = 0.04, β = 0.14), COVID-19-related deaths in the family (*P* = 0.02, β = 0.14), and social isolation (*P* = 0.03, β=-0.13) were associated with COVID-19 anxiety among older adults.

**Conclusion:**

The findings of this study indicated that social isolation can predict COVID-19 anxiety in older adults. It is expected that policymakers and family members of older adults will take steps to enhance community-oriented approaches, such as group meetings and face-to-face visits, while adhering to the relevant health protocols.

## Background

COVID-19, characterized by acute respiratory syndrome, is one of the most significant diseases that has emerged in the current century and continues to persist [1]. This acute respiratory illness has rapidly spread worldwide due to its high transmissibility, infecting all countries within less than four months [2]. In response to this disease, the World Health Organization has issued several public health recommendations, governmental measures, social distancing guidelines, and restrictions [3]. Although these restrictions help reduce the spread of infection, limiting participation in daily activities, physical exercise, and travel also have negative consequences [4]. One of the vulnerable groups during this pandemic is the elderly [5].

Elderly individuals are more significantly affected by COVID-19 due to underlying health conditions, resulting in a higher risk of mortality than younger populations [6]. Furthermore, older adults are at greater risk of psychological disorders due to reduced reserve capacity, the deterioration of bodily systems, multiple health issues, and diminished physical and financial independence compared to other adult age groups [5]. Social isolation is one factor that generally has a high incidence in this age group [7]. However, measures such as social distancing reduce social interactions and increase isolation among these individuals [8].

Social isolation is defined as the physical and social separation of individuals from one another, resulting in the breakdown of relationships, limited communication, withdrawal from social interactions, and a shrinking social circle around the individual [9]. Social isolation among the elderly is a serious concern, with its prevalence among individuals over 60 years reported to be between 24% and 7% [10]. Social isolation is one of the challenges of the modern era, particularly affecting older adults and it significantly impacts the health and quality of life of many elderly individuals. Given the effects of social isolation on the health of older adults and the forced physical distancing during the COVID-19 pandemic, it may have a more pronounced impact on their health than other demographic groups [11, 12]. Okrzac et al. also demonstrated that social distancing could increase loneliness and isolation within the community, particularly among the elderly [13]. However, some studies have reported contradictory results. Van Tilburg et al. found that social distancing did not result in social isolation within the community [14]. Nevertheless, social isolation can affect the physical and mental health of older adults, potentially leading to issues such as depression and anxiety [8, 15].

Anxiety is one of the most common psychological symptoms during the outbreak of COVID-19 and similar diseases [16]. COVID-19 anxiety refers to the anxiety arising from the fear of contracting the coronavirus [17]. It is associated with feelings of fear and ineffective anxiety related to the COVID-19 crisis. Factors such as the unknown nature of COVID-19, ongoing genetic changes, difficulties in diagnosis, and its rapid transmissibility are considered contributors to COVID-19 anxiety in the community, particularly among the elderly [18].

However, studies indicate that COVID-19 anxiety is related to various variables, including the sense of coherence, stress, social support, feelings of loneliness, and social isolation among the elderly [17]. Aghajani et al. noted that one of the factors influencing COVID-19 anxiety is social isolation [19]. Additionally, Millman et al. demonstrated that social isolation was associated with COVID-19 anxiety [20].

Overall, the elderly population is increasing both globally and in Iran. The prevalence of COVID-19 has altered social and economic structures, as well as the quality of relationships between older adults and other members of society [21]. On one hand, the fear of COVID-19 and the resulting social isolation have exposed this group to various psychological problems. Another noteworthy point is that older adults themselves, due to reduced social interactions and accompanying health issues, are considered a high-risk group for contracting illnesses. Although numerous studies have been conducted on COVID-19, there is currently a lack of information regarding the predictive role of social isolation in COVID-19 anxiety among older adults. However, providing necessary services and care for this group depends on identifying the variables influencing their psychological well-being. Given the insufficient information in this area and the cultural and contextual differences across various parts of the world, the present study was conducted to determine the predictive role of social isolation in COVID-19 anxiety among older adults.

## Methods

### Study design

This cross-sectional study with a descriptive-analytical approach was conducted in 2023 in Iran. The inclusion criteria for the study were residency in Qazvin City, age 60 years and older, willingness to participate in the study, and the absence of a confirmed psychological disorder.

Incomplete questionnaire responses or refusal to continue participation were considered exclusion criteria.

### Sample size

The sample size was calculated using power analysis and the G Power software to ensure sufficient statistical power for the test. Assuming a type I error of α = 0.05 and an effect size of f2 = 0.05, this study required a sample size of 309 to identify significant regression coefficients with a power of 80%.

The study population consisted of all individuals aged 60 and older in public places, including parks and mosques. Qazvin City was divided into three districts, each considered a cluster.

From each cluster of parks and mosques, participants were randomly selected to enter the study. Older adults from these mosques and parks who had electronic health records at comprehensive health service centers were included in the study if they met the inclusion criteria.

### Data collection

In order to collect data, several questionnaires were used, including the Demographic Information Questionnaire, the COVID-19 Anxiety Questionnaire, and an additional questionnaire.

The Demographic Information Questionnaire included information regarding age, gender, religion, marital status, number of children, educational level, occupation, income, economic status, type of residential house, living status with family members, insurance status, presence of COVID-19-infected individuals among relatives, death of relatives due to COVID-19, history of physical and mental illness, and hospitalization history.

Ten professors in nursing education, gerontology, and geriatric nursing reviewed the Demographic Information Questionnaire, and their feedback and opinions were incorporated to establish content validity,

The validity and reliability of the COVID-19 Anxiety Scale (CDAS) were examined and confirmed by Ahmad Ali Pour et al. in the Iranian sample in 2019. This questionnaire consists of 18 items using a 4-point Likert scale, with scores ranging from “Never = 0” to “Sometimes = 1,” “Often = 2,” and “Always = 3”. The Cronbach’s alpha coefficient was calculated to assess internal consistency, resulting in 0.872 for psychological symptoms, 0.860 for physical symptoms, and 0.820 for the overall questionnaire.

The scores of the COVID-19 anxiety scale were categorized into three domains: no or mild anxiety, moderate anxiety, and severe anxiety. In the preliminary validation, the COVID-19 Anxiety Scale demonstrated good validity and reliability, making it a reliable and scientifically valid tool for measuring anxiety related to the COVID-19 virus [22].

The Lubben Social Network Scale (LSNS) is a questionnaire designed to measure social isolation in older adults. It consists of 12 items using a 5-point Likert scale. Higher scores indicate higher levels of social support and lower social isolation. Scores below 20 indicate a severe risk of social isolation [23]. In a study by Rahmani et al., the reliability of this questionnaire was confirmed [24].

### Data analysis

Data analysis was performed using SPSS software version 23. Descriptive mean and standard deviation tests were used for continuous quantitative variables, such as age. Besides, frequency and percentage were used for categorical/nominal variables, such as gender and marital status. Statistical analysis was conducted to examine the predictive power of independent variables on COVID-19 anxiety using a multiple regression model, and a significance level of *p* ≤ 0.05 was considered.

## Results

The study sample consisted of 300 older adults, with a mean age of 71.04 years (SD = 9.88), ranging from 60 to 99 years old. Most participants, 177 older adults (59.0%), were female, and 225 (75.0%) were married. Table 1 provides the information about the demographic characteristics of older participants.

**Table 1:** Demographic characteristics of the older adults (*n* = 300)

Variable		*N*	Percentage
Gender	Male	123	41.0
	Female	177	59.0
Marital Status	Married	225	75.0
	Single	20	6.7
	Divorced	15	5.0
	Widow	40	13.3
Educational level	Illiterate	128	42.7
	Primary	79	26.3
	Secondary and diploma	55	18.3
	Academic	38	12.7
Living status	With spouse	93	31.0
	With spouse and children	120	40.0
	With children	32	10.7
	With others	9	3.0
	Alone	46	15.3
Job	Housewife and unemployed	166	55.3
	Employed	76	25.3
	Retired	58	19.3
Financial status	Low	37	12.3
	Middle	163	54.3
	Good	87	29.0
	Excellent	13	4.3
Children	0	16	5.3
	1–3	115	38.3
	4–5	85	28.3
	5 ≤	84	28.0
COVID-19 infection in family	Yes	166	55.3
	No	134	44.7
COVID-19 death in family	Yes	122	40.7
	No	178	59.3
Chronic disease history	Yes	218	72.7
	No	82	27.3

The mean, standard deviation of COVID-19 anxiety and social isolation among older participants were 10.99 (9.10) and 28.10 (7.89), respectively

**Table 2:** Predictors for anxiety related to COVID-19 among older adults during COVID-19 pandemic

Variables		Crud β Coefficient (CI: 95%)	Adjusted β coefficient (CI: 95%)
Social Isolation		**-0.19(-0.35, -0.09)** ***P*** **= 0.001**	**-0.13(-0.28, 0.10)** ***P*** **= 036**
Age		**0.14(0.03**,** 0.23)** *P* = 0.015	0.01(-0.11, 0.13) *P* = 870
Gender		-0.05(-3.08, 1.13) *P* = 0.363	-
Marital status	Married	1	1
	Single	**1.62(1.77**,** 1.04)** *P* = 0.005	**0.14(0.23**,** 9.78)** *P* = 0.040
	Divorced	0.01(-4.29, 5.17) *P* = 0.855	-0.09(-8.78, 1.48) *P* = 0.163
	Widow	0.08 (-0.96, 5.12) *P* = 0.179	-0.03(-4.83, 3.14) *P* = 0.677
Level of education	Illiterate	1	1
	Elementary	**-0.12(-5.04**,** 0.06)** *P* = 0.055	-0.12(-5.11, 0.01) *P* = 0.05
	Secondary	-0.05(-4.11, 1.63) *P* = 0.395	-0.09(-5.07, 1.02) *P* = 0.191
	Academic	**-0.14(-7.09**,** -0.51)** *P* = 0.024	-0.13(-7.12, 0.29) *P* = 0.070
Economic situation	Low	0.08(8.99, 11.80) *P* = 0.215	-
	Average	**1**	**-**
	Good	0.04(-1.57, 3.19) *P* = 0.505	**-**
	Excellent	0.05(-2.87, 7.46) *P* = 0.383	**-**
Occupation	Housewife-unemployed	1	1
	Retired	**0.10(-3.00**,** 5.09)** *P* = 0.081	0.0.08(-1.08, 4.53) *P* = 0.226
	Employed	**-0.13(-5.09**,** -0.20)** *P* = 0.034	-0.06(-3.79, 1.48) *P* = 0.391
Living status	With children and spouse	1	1
	With spouse	0.04(-1.69, 3.22) *P* = 0.541	-0.05(-3.56, 1.43) *P* = 0.400
	With children	0.08(-1.28, 5.79) *P* = 0.210	0.05(-2.24, 5.41) *P* = 0.415
	Alone	**0.18(1.38**,** 7.54)** *P* = 0.005	0.12(-1.15, 7.03) *P* = 0.158
	Others	0.03(-4.48, 7.81) *P* = 0.593	-0.02(-7.48, 5.84) *P* = 0.809
Children	0	0.11(-0.25, 9.27) *P* = 0.063	-
	1–3	1	-
	4–5	0.10(-0.64, 4.46) *P* = 0.142	-
	More than 5	0.02(-2.23, 2.89) *P* = 0.802	-
COVID-19 death in family	Yes	**0.21(1.73**,** 5.86)** *P* < 0.001	**0.14(0**,**25**,** 3.00)** *P* = 0.020
Family COVID-19		0.00(-2.07, 2.10) *P* = 0.988	**-**
Chronic disease history	Yes	**0.18(1.29**,** 5.86)** *P* = 0.002	**0.12(0.05**,** 4.65)** *P* = 0.045

Note: β: Standard regression coefficient; SD: Standard Deviation; CI: Confidence Interval

The results of the multivariable regression model showed that marital status, COVID-19 death in the family, history of chronic disease, and social isolation were associated with COVID-19 anxiety in older adults (Table 2). The COVID-19 anxiety was increased by increasing the social isolation (β = -0.13, *p* = 0.036). Those with a history of chronic disease (β = 0.12, *p* = 0.045) and COVID-19 death in the family (β = 0.14, *p* = 0.020) reported higher COVID-19 anxiety. Furthermore, single older adults reported higher anxiety related to COVID-19 (β = 0.14, *p* = 0.040) compared with those who were married.

## Discussion

This study aimed to investigate the predictive role of social isolation in COVID-19 anxiety. The results indicated that social isolation was associated with COVID-19 anxiety in the elderly population. This finding is consistent with the study conducted by Milman et al. [20], where they also demonstrated that adherence to social isolation policies predicts COVID-19 anxiety. Siu et al. demonstrated a one-way effect of social isolation on anxiety symptoms among elderly adults, although anxiety did not appear to influence social isolation [25]. While this study did not specifically address COVID-19 anxiety, its results align with the current study. Additionally, the research conducted by Rob et al. at the onset of the COVID-19 pandemic revealed a significant and inverse relationship between subjective loneliness and anxiety [26] Although this study did not focus on COVID-19 anxiety and instead examined hospital-related anxiety, it is conceptually consistent with this study regarding the relationship between social isolation and anxiety.

These findings are consistent with Aghajani et al. [19], Holt-Lunstad et al. [27], and Patchana et al. [28], which also suggest that social isolation predicts COVID-19 anxiety. Moreover, during the quarantine period in Wuhan, China, emergency workers experienced increased rates of depression, anxiety, and post-traumatic stress disorder due to social isolation [29].

To explain these findings, we must consider two interrelated issues. On one hand, aging contributes to increased social isolation and loneliness among individuals. On the other hand, the restrictions imposed during the COVID-19 pandemic have also significantly impacted the rise in isolation and loneliness [30]. Therefore, aging and COVID-19 have collectively exacerbated the social isolation. This isolation can further deteriorate an individual’s mental health and contribute to heightened anxiety symptoms [25].

Although isolating older adults is essential for their physical safety, its implementation without proper management, supervision, and necessary support can worsen their mental health, jeopardize their independence, and increase their feelings of loneliness. Undoubtedly, distancing from society is an unpleasant experience for all individuals, especially older adults, and can lead to increased stress [31]. This situation exacerbates the condition for older adults who were already experiencing social isolation and may even lead to the emergence or intensification of stress and anxiety [31]. Given these explanations, the findings of the present study are justified.

The recent study demonstrated a significant association between marital status and COVID-19 anxiety in the elderly population, with unmarried elderly individuals experiencing higher levels of anxiety. This finding is supported by the study conducted by Janzen et al. [32], where marital status was associated with the average anxiety levels in diabetic patients. In contrast, Zare et al. [33] showed no association between anxiety and marital status in women, which is inconsistent with the results of the current study.

To explain this difference, it should be noted that this study was conducted among housewives with an average age of 35.7 years, whereas our study focuses on the elderly population. Individuals in young and middle adulthood generally possess greater self-management and self-care abilities than older adults. Aging is associated with numerous limitations that affect an individual’s capacity for self-care. Given that the participants in the study by Zare et al. were younger, it is possible that the influence of marital status on anxiety was impacted by their age, leading to differences in the results of the two studies.

In addition, the researcher believes that the issue of loneliness and its impact on anxiety should be addressed. The researcher argues that being single, especially in old age, and lacking companionship to talk to during times of need and stress can significantly affect an individual’s level of anxiety. Moreover, it may even contribute to the development of anxiety itself.

Our results showed that elderly individuals who had experienced the death of their relatives due to COVID-19 had higher levels of anxiety. This finding is supported by the study conducted by Rashti et al. [5], where elderly individuals who had experienced COVID-19 deaths in their families were more likely to suffer from COVID-19 obsessive thoughts and anxiety compared to those who did not experience any deaths among their relatives.

During a pandemic caused by a disease like COVID-19, the fear of illness and death, coupled with disruptions to daily activities, can lead individuals to experience illness-related anxiety [34]. The researcher posits that when a relative dies from an unknown emerging disease, it can significantly heighten anxiety about contracting COVID-19. Therefore, it is essential for families and other relatives to prioritize the mental health care of older adults, as this requires special attention.

Furthermore, it should be noted that the prevalence of the pandemic and the resulting physical separation exacerbate feelings of sadness among family members. If a family member passes away due to illness, this can expose the remaining family members to various issues, such as depression and anxiety [35]. Therefore, it is not surprising that the findings of the present study indicate that the death of a relative can play a role in predicting COVID-19 anxiety.

The mortality rate of COVID-19 is higher in elderly individuals with underlying diseases, and studies have shown that COVID-19 infection is more common and severe in elderly individuals with conditions such as diabetes, cancer, and kidney diseases [36]. In this study, there was a significant difference in COVID-19 anxiety scores between elderly individuals with at least one underlying disease and those without any underlying diseases. Rashti et al. [5] and Azmir et al. [37] also demonstrated a significant association between underlying diseases and COVID-19 anxiety. Alizadeh et al. [38] also showed a significant association between anxiety disorders and underlying diseases among the elderly population. Data on COVID-19 patients indicate that individuals of all age groups are susceptible to infection; however, the weakening of the immune system and the prevalence of underlying health conditions in the elderly exacerbate the consequences of this disease, resulting in a higher mortality rate. Specifically, the mortality rate from COVID-19 among older adults and those with pre-existing health conditions has been reported to be greater than in other age groups [5] This situation may further increase psychological problems and anxiety among the elderly.

The researcher, who also has experience working in various hospital departments, believes that having underlying health conditions and concurrently taking multiple medications in older adults affects their levels of anxiety and fear of contracting COVID-19.

The association between anxiety levels and chronic diseases during the COVID-19 pandemic may be due to the emphasis on the higher mortality rate in COVID-19 patients with underlying diseases in various media sources.

Our results showed that the level of COVID-19 anxiety in the elderly population was not significantly associated with age, gender, education level, or economic status. These findings are consistent with the study by Zare et al. [33], which also showed that age and education level were not related to anxiety levels. However, they contradict the study by Janzen et al. [32], who demonstrated a significant statistical relationship between age and COVID-19 anxiety in diabetic patients, inconsistent with our results.

In the study conducted by Ozarin et al. [37], contrary to this study, gender, specifically being female, was identified as a risk factor for COVID-19 anxiety. However, in this study, there was no significant association between the level of anxiety in the elderly population and gender. It appears that in the elderly population, gender may not be an associated factor for anxiety and stress, and both male and female may tolerate similar levels of anxiety.

## Limitations

The small sample size and cultural, social, and contextual differences pose limitations on the generalizability of the results. Another limitation of the study was the low willingness of elderly participants to engage. However, this limitation was somewhat mitigated by providing ample time and opportunities, as well as clearly explaining the study’s objectives and rationale.

## Conclusion

The results indicate that social isolation is associated with anxiety and stress in the elderly population. Additionally, the findings indicated that marital status and the death of relatives following COVID-19 infection play significant roles in COVID-19 anxiety. Specifically, being single and experiencing the death of a relative are associated with increased anxiety levels among the elderly. Therefore, considering the high prevalence of COVID-19, addressing these two variables in educational and cognitive workshops could be effective in reducing COVID-19 anxiety.

Policymakers and family members of these individuals are expected to create conditions that allow them to engage more with one another through face-to-face meetings and group sessions while adhering to the relevant protocols for preventing COVID-19 infection. Health policymakers can also include educational programs aimed at improving the psychological well-being of the population, with a focus on controlling social isolation. They can educate the elderly on self-care behaviors to combat social isolation.

## Data Availability

The datasets used and/or analyzed during the current study available from the corresponding author on reasonable request. The entire dataset is in Farsi language. The Data can be available in English language for the readers and make available from the corresponding author on reasonable request.

## Abbreviations

COVID-19: Coronavirus disease 2019
LSNS: Lubben Social Network Scale
CDAS: COVID-19 Anxiety Scale
